# Losing seasonal patterns in a hibernating omnivore? Diet quality proxies and faecal cortisol metabolites in brown bears in areas with and without artificial feeding

**DOI:** 10.1371/journal.pone.0242341

**Published:** 2020-11-12

**Authors:** Agnieszka Sergiel, Isabel Barja, Álvaro Navarro-Castilla, Tomasz Zwijacz-Kozica, Nuria Selva

**Affiliations:** 1 Institute of Nature Conservation, Polish Academy of Sciences, Krakow, Poland; 2 Department of Biology, Universidad Autónoma de Madrid, Madrid, Spain; 3 Centro de Investigación en Biodiversidad y Cambio Global (CIBC-UAM) Universidad Autónoma de Madrid, Madrid, Spain; 4 Tatra National Park, Zakopane, Poland; Universidad de Guadalajara, MEXICO

## Abstract

Bears are omnivores particularly well-adapted to variations in the nutritional composition, quality and availability of food resources. Artificial feeding practices have been shown to strongly influence diet composition and seasonality, as well as to cause alterations in wintering and movement in brown bears (*Ursus arctos*). In this study, we investigated seasonal differences (hypophagia vs hyperphagia) in food quality of two brown bear subpopulations in the Polish Carpathians using faecal nitrogen (FN) and carbon (FC) estimates. The subpopulations inhabit areas that differ in artificial feeding practices: no artificial feeding occurs in the western subpopulation (Tatra Mountains), while artificial food targeted to ungulates is provided and used year-round in the eastern subpopulation (Bieszczady Mountains). We also compared these results with faecal cortisol metabolites (FCM) to explore how FN and FC correlate with the hypothalamic-pituitary-adrenal axis activity and if the seasonal patterns are apparent. We found that in Tatra Mts bears fed on significantly higher quality diet, as shown by FN and FC values, and had significantly higher FC levels in hyperphagia, when they accumulate fat reserves for wintering. The pattern in FCM levels for Tatra subpopulation followed the changes in energy intake during the seasons of hypo- and hyperphagia, while in Bieszczady Mts, the area with intensive feeding, no seasonal patterns could be observed. Artificial feeding practices may disrupt nutrient phenology and seasonality, relative to subpopulations with natural diets. We showed that the availability of human-provided foods may alter not only the overall dietary quality, but also hormonal patterns linked to seasonal nutritional requirements. Combining FN, FC and FCM proved to be a useful tool for reconstructing diet quality and related physiological patterns.

## Introduction

An explicit evaluation of the nutritional parameters of an animal’s diet is essential to comprehend foraging patterns in different habitats and ecosystems [1,2]. Faeces are the most accessible biological product from wildlife in nature, and their analysis is one of the most commonly used techniques for assessing food habits and dietary quality in mammals [3–6]. These studies are no longer limited to the identification of undigested food remains. A strong understanding has been developed on the relationship between elemental composition of mammal faeces and their diet, and nitrogen and carbon faecal concentrations have become proxies of diet quality [7,8]. The protein content of forage and its digestibility are directly related, and a high correlation between the chemical composition of forages and nitrogen excreted in faeces (faecal nitrogen, FN) has been experimentally proved [9]. Seasonal and spatial variations of FN are found in wild large herbivore populations, with values peaking in spring in response to higher digestibility and protein content in growing vegetation [10–12]. A similar pattern has been observed in primates [13,14] and rodents [15,16], and several free-ranging omnivore species [17]. The percentage of nitrogen content in faeces represents the protein content of the food consumed, and, thus, it is considered as a biomarker for diet quality and shifts in diet composition [7,8,13,18], while faecal carbon (FC) indicates changes in energy consumption and shifts in energy intake from digested food items [7].

Both energy intake and storage are regulated by glucocorticoids, which have an additional role in the physiological stress response [19,20]. The urge to eat is a complex process regulated at many levels [21], but glucocorticoids are key players stimulating the appetite and influencing the type of food to seek for [22]. Cortisol, a primary glucocorticoid in most mammal species, is also involved in maintaining the balance between fat storage and fat depletion [23]. As glucocorticoids mediate redistribution of energy into different types of fat and regulate glucose levels in synergy with insulin [24,25], it has been hypothesized that they play a major role in regulating hyperphagia and autumn fat storage required in hibernating species. Seasonal pattern of glucocorticoids would, then, show an increase during hyperphagia to promote foraging behavior and, thus, increase the energy input and storage [24]. Romero et al. [26] tested this hypothesis on few rodent species and found that glucocorticoid concentrations were lower in the fall in species relying upon endogenous fat reserves during hibernation, but higher in species that cache food to survive winter. Nevertheless, most studies provide clear evidence that, although following different patterns, a majority of reptilian, amphibian, avian, and mammalian species seasonally modulate glucocorticoid concentrations [27].

Brown bears (*Ursus arctos*) are hibernating omnivores whose diet varies geographically and seasonally across its range [3,4,6]. Bears consume mostly plants [e.g. 28–30] and their diets largely reflect changes in plant phenology [e.g. 31,32]. They also track seasonal or inter-annual pulses of high-calorie resources, e.g. hard and soft mast in interior populations [5,31] or salmon in coastal ecosystems [28,33,34]. The availability of foods with high content in lipids or carbohydrates, necessary to maintain a balanced energy intake, co-occurs with the prehibernation hyperphagic period in bears during late summer and autumn [35]. The increased provision of human foods (in ecosystems), both intentionally and unintentionally, is changing these patterns and is of increasing concern [36]. Artificial feeding alters the spatio-temporal availability of natural foods and recent studies have evidenced changes in brown bear diet composition and seasonality due to this practice [37–39], as well as alterations of energy-related activities such as wintering [40], and movement [41]. Diet quality is an essential driver of individual performance, and therefore, drives population dynamics and has important effects on density, dynamic and life history of populations [42,43]. On the top of it, human provisioning of food can cause changes in the nutritional ecology and physiology of wildlife, which are typically negative and largely impact foraging behavior and food availability [44]. The extent to which changes in food availability affect the fitness of organisms is not clearly understood and requires different approaches. The presence of areas where food provision to wildlife is prohibited and where such practice is common, constitute natural experiments that should contribute to improve our understanding of the role of food supply in a range of ecophysiological processes related to nutrition and stress in wildlife. Artificial feeding is associated to negative ecological effects [see 45 for review], in addition to the alteration in diet composition, wintering and movements reported for bears mentioned above. Anthropogenic food of low quality may also compromise wildlife condition and immune function [46]. As provisioning occurs in many different contexts around the world [36] it may have important consequences for species of conservation concern.

The digestive efficiency of bears is similar to those of obligate carnivores, meaning they can process only highly digestible nutrients [47,48]. FN levels have been shown to vary directly with dietary nitrogen intake in black bears (*Ursus americanus*) [47,49]. Although some studies indicate strong correlation of glucocorticoids and dietary indices of excreta in other mammal species [7], to our knowledge no study has used the combined approach of FN and FC and hormonal analysis of faeces (faecal cortisol metabolites, FCM) to investigate seasonal patterns in food quality in bear populations. Using the brown bear as a model species of a seasonal mammal, we investigated whether the association between diet quality proxies (FN, FC) and glucocorticoids levels (FCM) and their seasonal patterns are affected by artificial feeding practices. We examined seasonal variation in FN, FC and FCM in the two core areas of the brown bear population in the Polish Carpathians, which differ in the management practices regarding artificial feeding. We hypothesized that if a link exists between diet quality (assessed with FN and FC) and cortisol, then differences in cortisol metabolite concentrations in faeces would be associated with dietary differences between the study areas. It could be expected that the subpopulation foraging on human-provided food should have lower FCM values because consumption of abundant and year-round available resources should confer nutritional benefits (higher FC and FN) resulting in lower nutritional stress. However, if nutritional requirements are met in both core subpopulations, then we would expect no differences in FCM associated with dietary differences. Additionally, we tested the hypothesis that the use of year-round available resources of artificial foods provided by humans with constant nutritional gain would disrupt natural seasonal patterns in diet quality and glucocorticoids levels. We also explore whether faecal nutritional biomarkers combined with FCM data might be a worthwhile approach to gain insight into the dietary and related hormonal patterns.

## Materials and methods

### Study areas

**Fig 1:**
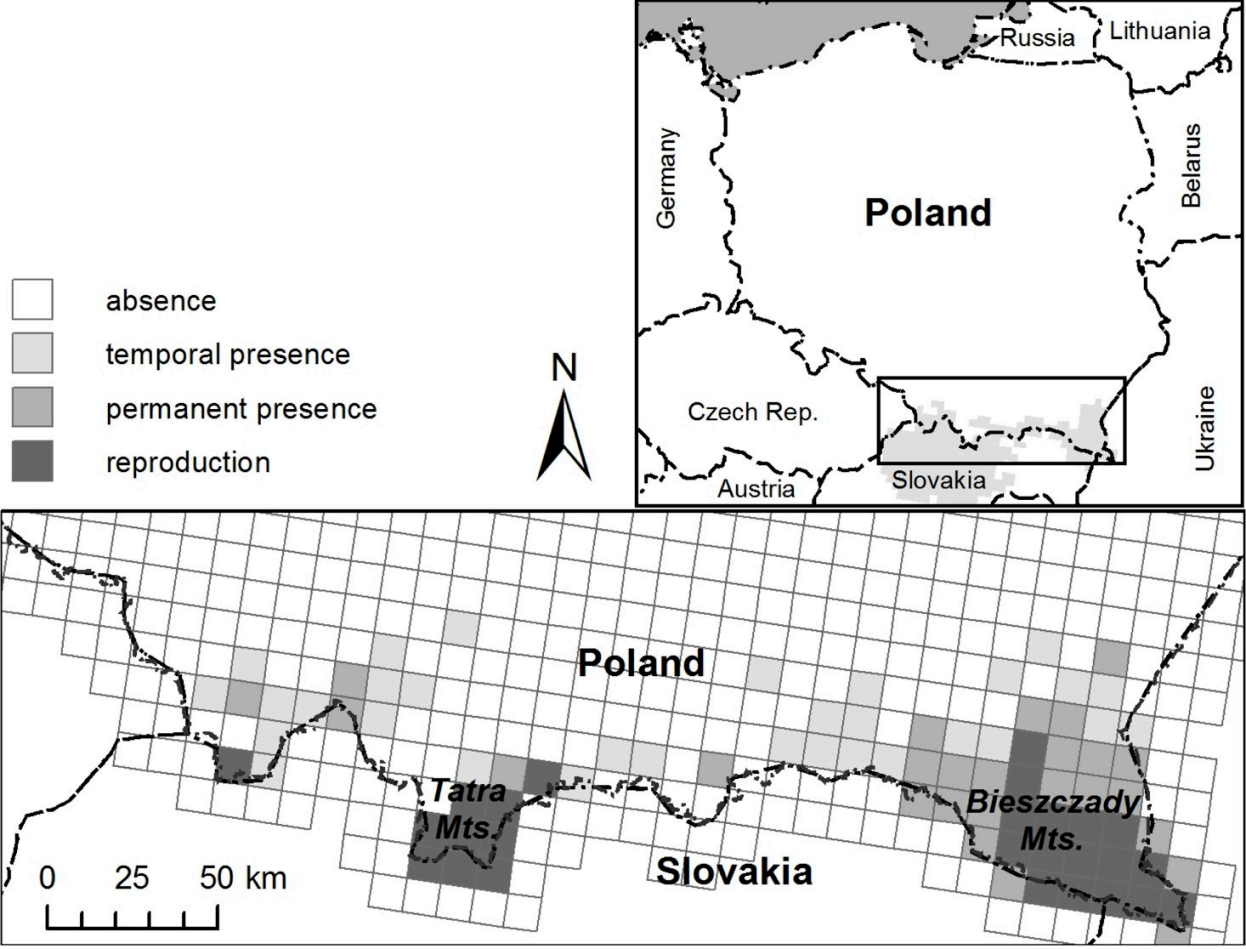
Study areas in Tatra and Bieszczady Mts with current bear distribution shown on 10x10 km grid.

The study was conducted in the Polish Carpathians, in the western and eastern core areas inhabited by brown bears, Tatra and Bieszczady Mts, respectively. The Polish part of the Tatra Mts (Fig 1), with altitudes ranging from 790 to 2499 m a.s.l., is entirely included within the Tatra National Park (19,95°E; 49,25°N). Snow cover lasts for about 100 days in lower parts and up to 290 days on the tops. The landscape is of alpine type with mean annual temperatures 2.4 and 5.4°C at the timberline (*i*.*e*. 1500 m a.s.l.) and the lowest elevations, respectively. Precipitation increases with elevation and averages 1100 mm for the lowest elevations and 1700 mm for the timberline. Typical vegetation includes Norway spruce (*Picea abies*), the dominant tree species in the area. Up to 1250 m a.s.l., it grows in mixtures with European beech (*Fagus sylvatica*) and silver fir (*Abies alba*). Meadows are scattered throughout the forest. The number of visitors is very high (approx. three million per year), but human activities are restricted to designated areas and trails, and no artificial feeding is conducted [50]. The habitat of Tatra Mts supports brown bear occurrence, reproduction and movement and the number of bears inhabiting the area has been estimated in 45–79 individuals [51,52].

The second study area, located in the Bieszczady Mts (SE Poland, Fig 1), is characterized by gentle slopes (maximum altitude 1350 m a.s.l.) and dominated by beech and fir [53]. The annual average air temperatures range between 3–8°C, depending on the latitude and climatic zone, with the annual average precipitation of 900–1,300 mm [54]. At altitudes within 500–800 m a.s.l., the snow cover persists for 90–140 days [55]. The forest is intertwined with meadows, particularly in the lower parts of the mountains, and represents a suitable habitat for brown bear occurrence, reproduction and movement [53,56]. Most of this study area is commercially managed by the Polish State Forest Holding for timber extraction and game hunting, except the Bieszczady National Park. The number of visitors is significantly lower than in Tatra, about 500 thousand people a year (in the Bieszczady National Park; [57]). Artificial food (corn, beetroots, fodder, grain) targeting at ungulates, mostly red deer (*Cervus elaphus*), bison (*Bison bonasus*) and wild boar (*Sus scrofa*), is regularly provided, usually at the same locations (feeding sites) and practically year-round, with an increase at the end of summer throughout early spring. Numerous target and non-target species use this human-provided food intended for ungulates [58], and brown bear movement in the area is significantly affected by feeding practices [41]. Brown bears inhabiting the area are estimated at 46–61 individuals and belong to the eastern Carpathian subpopulation [51].

### Faecal sample collection

We used a set of 142 faecal samples from the two brown bear subpopulations inhabiting the two core areas from 2008 through 2011 during the bear activity period. Only fresh bear scats, i.e. less than 48 hours old, were collected [59]. We assessed scat freshness based on the smell, color and appearance of the scat, bear signs, weather conditions and our field experience with inspections of GPS fixes of collared bears. Samples were put directly into sterile tubes, labeled, transported in an insulated bag with frozen gel packs and stored at -20°C until laboratory analysis. We noted the date and hour of collection, GPS location, and used blind observation methods by encoding collected samples before laboratory analysis of FN, FC and FCM concentrations.

### Ethics statement

The field work was conducted in accordance with legal requirements in Poland. Apart of the restricted access area of the Tatra National Park, where the samples were collected by National Park staff (TZK) under routine conservation and monitoring tasks, sample collection was conducted in the public forest lands, managed by the Polish State Forest Administration. Collection of faecal samples in public state forest, being non-invasive, does not require any permit.

### Analysis of nutritional biomarkers: FN and FC

Faecal samples were unfrozen and dried in a heater (24 h at 90°C) to eliminate water content. Faeces were manually homogenized and subsamples were collected from each scat to determine FN and FC. Dried faeces were mixed with 10ml of liquid nitrogen to completely pulverize the sample with a mortar and a pestle. Later, ca. 1g of each pulverized sample was stored in a properly labeled tube and sent to the Research Support Central Services (SCAI) laboratory at the University of Málaga (Spain) for analysis. Quantification of FN and FC was carried out by means of the elementary chemical analysis following the classical Pregl-Dumas method [60,61]. The technique was performed on a PERKIN-ELMER 2400 CHN elemental analyzer. Samples were measured and processed taking into account the weight of the sample as well as the data provided by a standard sample, thus obtaining the content of each element (i.e. N and C) in the sample. The results of FN and FC are presented as percentage (%) of dry matter.

### Extraction and quantification of FCM

Faecal samples were unfrozen and dried in the heater until constant weight (24 h at 90°C). Rapid defrosting by heating is proved to keep FCM stable and concentrations measured in dried faeces are comparable to those obtained from wet faecal samples [62,63]. Later, 0.5 g of dry faeces were weighted and placed in assay tubes with 2.5mL of phosphate buffer and 2.5mL of pure methanol. Tubes were vortexed and then shaken for 16 h. Afterwards, supernatants were centrifuged (4000 g 30 min) and the resulting faecal extracts were stored at -20°C until analysis.

Quantification of FCM was carried out using a commercially available cortisol enzyme immunoassay kit (Cortisol ELISA DE1887; Demeditec Diagnostics GmbH,Kiel, Germany). Validation of the EIA used was performed by carrying out parallelism, accuracy and precision tests [64]. Parallelism was performed with serial dilutions of faecal extracts (1:32, 1:16, 1:8, 1:4, 1:2, 1:1) resulting in a curve parallel to the curve built with the standards provided in the kit. Accuracy (recovery) was 89.3 ± 8.9%. Precision was tested by means of intra- (8.6%) and inter- (10.5%) assay coefficients of variation. According to the manufacturer, sensitivity (detection limit) of the assay was 2.5 ng/ml. Cross-reactivity values of this assay for cortisol, corticosterone, progesterone, deoxycortisol and dexamethasone are 100, 45, <9, <2 and <2%, respectively, and <0.01% with estriol, estrone and testosterone. FCM concentrations are expressed as ng/g of dry faeces.

### Statistical analyses

To properly interpret the quality of food patterns and the seasonal variations, we considered two foraging periods (hypophagia and hyperphagia), typical in the brown bear annual cycle, and assumed that foraging starts when bears leave their winter dens in early spring. This first period is characterized by lower food intake (hypophagia) [65]. In the Bieszczady Mts bear activity is observed in winter and food intake also occurs then, particularly at ungulate feeding sites [38,41,66]. As we were interested in the effects of food intake, we treated winter, spring and early summer within the hypophagic period (January-July). With summer progressing, the food intake increases markedly (hyperphagia; [67]) until the bears gain enough body mass before denning [25]. Denning takes place mostly from November to April in Tatra mountains [51], whereas in Bieszczady, some bears are active during winter, partly due to artificial feeding [66]. Thus, samples span the active period, which allow us to examine temporal patterns in the nutritional quality of bear diets. We did not consider inter-annual variations.

We used generalized linear models (GLMs) with gaussian (FN, FCM) and gamma (FC) distribution errors to explain the variation in the response variables (FN, FC and FCM). FCM and FN data were log- transformed to achieve better model fit. We used as explanatory variables the season (hypo- and hyperphagia), area (Bieszczady and Tatra Mts) and the interaction between them. We combined hypothesis testing and information theory approaches to maximize the understanding of our system [68]. We also investigated how FCM levels (log-transformed) were affected by food quality (FN, FC) using GLMs with a gaussian error distribution and identity link function. Relationship between diet quality indices was investigated with Pearson’s correlation test. The analyses were performed in R 3.6.0 [69].

## Results

In total, 142 samples were analyzed, including 57 and 85 samples for subpopulations in Tatra and Bieszczady Mts, respectively. FN and FC were obtained for 98 samples. The values of FN, FC and FCM are summarized separately for the two study areas in Table 1.

**Table 1:** Values of faecal nitrogen (FN, in % of faecal dry matter), faecal carbon (FC, in % of faecal dry matter) and faecal cortisol metabolites (FCM, in ng/g of faecal dry matter) in brown bear faeces, collected in Tatra and Bieszczady Mts in Poland in 2008–2011.

	Tatra Mts	Bieszczady Mts
FN (%, mean ± SD)	2.475 (±0.532)	1.826 (±0.567)
FC (%, mean ± SD)	48.255 (±5.235)	43.887 (± 5.776)
FCM (ng/g, mean ± SD)	2568.346 (±3127.153)	616.092 (±328.166)

### Diet quality and FCM levels in study areas

**Fig 2:**
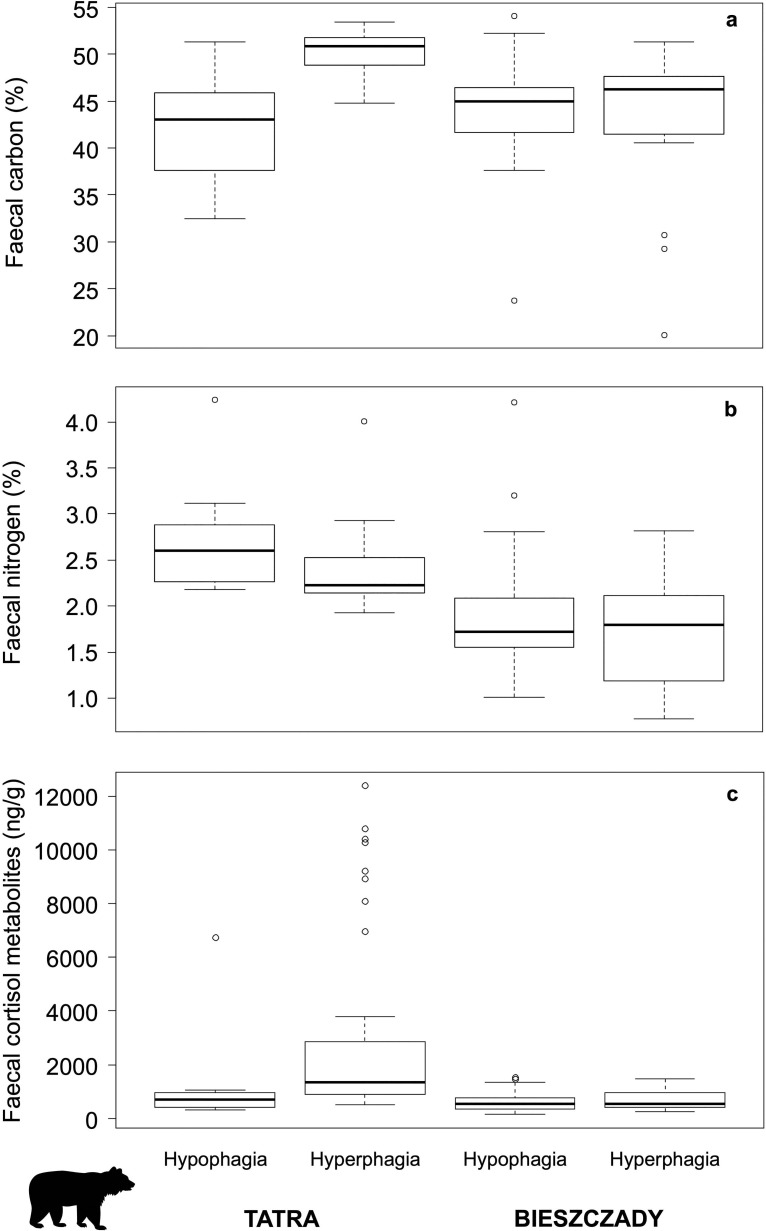
Faecal nitrogen (FN, %), carbon (FC, %) and cortisol metabolite (FCM, ng/g) values in the study areas.

Area was a significant explanatory variable for diet quality (Table 2). Both FN and FC were significantly higher in Tatra than in Bieszczady Mts (Tables 1 and 2, Fig 2). Regarding FCM, significantly higher levels were also found in Tatra Mts (Tables 1 and 2, Fig 2).

**Table 2:** Summary of model selection explaining the variation in faecal nitrogen (FN, %), faecal carbon (FC, %) and faecal cortisol metabolites (FCM, ng/g) in relation to the study area (reference category: Bieszczady Mts) and season (reference category: hyperphagia). We also provide the percentage of deviance explained by each model and the p-values of the predictors. Models were fitted using identity link and gaussian distribution (FN(%), FCM(ng/g)) and log link and gamma (FC(%)) error distributions. FN and FCM values were log-transformed.

Models	df	Explanatory variables	Estimate ± S.E.	P	Deviance explained (%)	AIC	Delta	AIC weight
FN ~ Area + Season	4	(Intercept)	0.481 ± 0.056	1.39e-13	26.9	23.6	0.00	0.502
		Area	0.383 ± 0.066	8.68e-08				
		Season	0.106 ± 0.062	0.0895				
FN ~ Area	3	(Intercept)	0.558 ± 0.033	< 2e-16	24.6	24.6	0.99	0.306
		Area	0.329 ± 0.059	2.04e-07				
FN ~ Area * Season	5	(Intercept)	0.488 ± 0.063	1.12e-11	26.9	25.5	1.93	0.191
		Area	0.370 ± 0.084	2.92e-05				
		Season	0.096 ± 0.074	0.196				
		Area*Season	0.035 ± 0.137	0.800				
FN ~ Season	3	(Intercept)	0.695 ± 0.048	<2e-16	0.0	51.3	27.70	0.000
		Season	-0.062 ± 0.063	0.328				
FC ~ Area * Season	5	(Intercept)	3.758 ± 0.029	2 e-16	17.7%	636.5	0.00	0.916
		Area	0.158 ± 0.038	8.27 e-5				
		Season	0.032 ± 0.033	0.334				
		Area*Season	-0.210 ± 0.062	0.00107				
FC ~ Area	3	(Intercept)	3.782 ± 0.015	< 2e-16	9.1	642.3	5.76	0.051
		Area	0.095 ± 0.027	0.000805				
FC ~ Area + Season	4	(Intercept)	3.802 ± 0.026	<2e-16	9.8	643.6	7.08	0.027
		Area	0.080 ± 0.031	0.0111				
		Season	-0.027 ± 0.029	0.3523				
FC ~ Season	3	(Intercept)	3.849 ± 0.020	<2e-16	4.9	646.7	10.22	0.006
		Season	-0.065 ± 0.026	0.0137				
FCM ~ Area * Season	5	(Intercept)	6.386 ± 0.138	< 2e-16	37.0	315.2	0.00	0.665
		Area	1.052 ± 0.172	9.83e-09				
		Season	-0.148 ± 0.168	0.3798				
		Area*Season	-0.622 ± 0.321	0.0549				
FCM ~ Area + Season	4	(Intercept)	6.501 ± 0.126	< 2e-16	35.2	317.0	1.81	0.270
		Area	0.873 ± 0.147	2.13e-08				
		Season	-0.317 ± 0.144	0.0299				
FCM ~ Area	3	(Intercept)	6.285 ± 0.080	< 2e-16	32.9	319.8	4.64	0.065
		Area	1.045 ± 0.126	8.24e-14				
FCM ~ Season	3	(Intercept)	7.064 ± 0.093	< 2e-16	18.7	347.2	31.97	0.000
		Season	-0.774 ± 0.136	7.65e-08				

There was a significant positive correlation between FN and FC (r = 0.270, df = 96, P = 0.007) indicating that higher protein food was accompanied by higher carbon food. Concentrations of FCM significantly increased with higher FN and FC values (Table 3). Food quality, expressed as FC and FN, explained 26% of the deviance in FCM.

**Table 3:** Summary of model selection explaining the relationship between faecal cortisol metabolites (FCM, ng/g) and food quality (faecal carbon FC and faecal nitrogen FN, %). Generalized linear models were fitted using identity link and gaussian error distributions. FCM values were log transformed.

Models	df	Explanatory variables	Estimate ± S.E.	P	Deviance explained (%)	AIC	Delta	AIC weight
FCM ~FC+FN	4	(Intercept)	3.140 ± 0.623	2.23e-6	25.9	236.9	0.00	0.559
		FC	0.065 ± 0.014	1.20e-5				
		FN	0.273 ± 0.132	0.0414				
FCM ~FC*FN	5	(Intercept)	4.222 ± 1.670	0.0131	26.3	238.4	1.49	0.265
		FC	0.041 ± 0.037	0.7035				
		FN	-0.338 ± 0.885	0.2672				
		FC*FN	0.013 ± 0.019	0.4865				
FCM ~FC	3	(Intercept)	3.339 ± 0.626	6.40e-07	22.6	239.2	2.31	0.176
		FC	0.073 ± 0.014	7.62e-07				
FCM ~FN	3	(Intercept)	5.735 ± 0.297	< 2e-16	9.3	254.8	17.88	0.000
		FN	0.439 ± 0.140	0.00232				

### Seasonal patterns in FN, FC and FCM levels

In Tatra Mts, FN showed higher values than in Bieszczady in both seasons and the interaction between area and season was not significant. When the interaction term was removed from the FN model, season became marginally significant, and FN levels tended to be higher during hypophagia in both areas (Table 2). For FC, the interaction was significant, and the model that included the interaction term was the best ranked (Table 2). The effect of season on FC was different between areas. While in Tatra FC values significantly varied between hypo and hyperphagia, no variation was observed in Bieszczady (Table 2, Fig 2). In the case of FCM, the interaction between area and season was close to significance and the full model was the best ranked. Variation in FCM levels between seasons was observed only in Tatra, but not in Bieszczady (Table 2, Fig 2). In Tatra Mts, minimum FCM value was 319,5 and maximum reached 6744,0 ng/g in hypophagia, and in range from 515,0 to 12411,3 ng/g in hyperphagia. In Bieszczady Mts, minimum and maximum FCM values were 136,0 and 1543,2 ng/g in hypophagia, and between 240,0 and 1461 ng/g in hyperphagia.

## Discussion

Our study shows that FC and FN can be useful nutritional indicators also for non-herbivorous species and provide useful insights into diet quality patterns. Their levels clearly showed that diet quality of brown bears differed between study areas. Significantly higher indices, both nitrogen and carbon, were observed in Tatra Mts, suggesting that bears forage on richer foods. A study on bear diet there showed graminoids and herbaceous plants are the most prevalent food source, and ants are the most important source of proteins (ca. 49, 29 and 22% frequency of occurrence, respectively). More importantly, no anthropogenic food items were found [70]. However, in Bieszczady Mts, where food quality markers showed lower levels, human-provided foods represent currently an important part of the bear diet, up to 80% in the winter months, and are used by bears year-round [41,71]. Earlier studies already identified corn (main food provided to ungulates) as the second most frequent food in spring (30.4% of the faeces) and the third in autumn (26.1%, [72]). Therefore, lower quality of the bear diet in Bieszczady Mts, as suggested by FN and FC levels, may be related to bears’ lifestyle being significantly affected by the artificial feeding practices in the area. These practices seem to be increasing in the amount of artificial food provided, number of feeding sites and duration of the feeding periods, not only in Poland, but generally in Europe and in particular, in the Carpathians [41,73,74].

Seasonally, FN values tended to be higher in hypophagia in both areas, but larger temporal variation was found in Tatra Mts. In Bieszczady, there were no apparent seasonal patterns in any of the diet quality indices, suggesting that bears were able to achieve relatively constant nutritional levels in their diet, but the overall diet quality was significantly lower than in Tatras. The difference in diet quality between study areas reflects variability in protein intake and food quality. Bears switch their diets towards protein and lipid-rich food, when available [3], and in general, they prioritize a protein intake target over carbohydrate or lipid, with a secondary preference of lipids over carbohydrates. In the absence of lipids, bears are able to utilize carbohydrates with equal efficiency–lipid and carbohydrate may serve as interchangeable non-protein-energy source when optimizing protein intake [29,35,75]. In the berry-rich areas, like the Tatra Mts, bears fatten in autumn by consuming large quantities of berries rich in carbohydrates [3] and optimize their macronutrient intake by mixing those high-energy fruits with all other foods (insects, ungulates, roots and green vegetation). In the absence of fruits, the diet becomes similar in macronutrient composition to the pregreen-up period [35,76]. In the study by Baldwin and Bender [47], although the diet of bears varied through seasons with an overall high quality, seasonal differences in FN were not recognized, which was accounted for a constant high protein level. Consistently, in the omnivorous chacma baboons (*Papio ursinus*) food of greater nutritional value (in terms of crude protein content) than the food of other savanna mammals was consumed throughout a year, optimizing nutritional uptake, resulting in higher FN values than in other mammal species in the same ecosystem and showing no apparent seasonal pattern [13]. In the Bieszczady Mts study area, although seasonal patterns were not observed, the FN levels indicate lower quality food sources (many of them human-provided) when compared to Tatra Mts. The effect of constant supplementary, low protein/high carbohydrates sources of food was addressed in the study by Landers et al. [77], which concludes that the diet must contain about 15% of crude protein for healthy growth of omnivores, including bears. However, Robbins et al. [29] demonstrated that protein content is not a minimum constraint on diet selection by brown bears. Corn contains about 9% of crude protein but is a rich source of nitrogen-free extract (concentrated energy) contributing to the overall nutrition. Fat, important for bears for survival while hibernating during winter, may be accumulated by the conversion of protein or carbohydrates to fat when the physiologically less expensive direct conversion of lipid from lipid is not possible due to shortages or seasonal unavailability of certain foods [3].

Glucocorticoids may be involved in the increased feeding and/or fat deposition characteristic of pregnancy and the pre-hibernation period in hibernating species [78]. We found overall FCM levels higher for the bears in Tatra study area, with lower values during hypophagia. However, it should be noted that a large portion of bear habitat in Tatra is within the area of influence of the dense touristic trail network, and that the high number of visitors might have additionally influenced FCM levels, as found for chamois (*Rupicapra rupicapra*) in Tatra [79]. This study showed that stress levels were significantly higher during the period with highest number of visitors, a result also found in other carnivore species living in protected areas [79–81]. Similar effects of tourism were found in other animal groups, including tetraonids, lagomorphs and elephants [82–84]. Nevertheless, the cortisol values still follow a seasonal pattern in Tatra, and the observed differences between study areas might be as well related to diet. In the study by von der Ohe et al. [85], FCM levels in brown bear samples containing flesh were lower than in samples made up of grass in months in which both were available (July and August). Samples from the berry diet type had lower FCM concentrations than those from the grass and flesh type. Overall, the high-fiber diet was demonstrated to result in higher faecal glucocorticoid levels in bears. Similarly, Stetz et al. [86] found that the main effects explaining variation in FCM concentrations in very fresh scats were food contents (vegetation vs. berries or meat), Julian date and aspect. Essentially, FCM were highest for scats containing primarily vegetation (presumably indicating relatively poor nutrition and/or high stress). Dietary fiber content might affect steroid excretion rates due to changes in gut transit time or faecal mass. Therefore, we dried samples prior to extraction, what has proved effective in hormonal analysis of faeces in other omnivores [87]. Fruits and vegetation contain high amounts of dietary fiber, but only faeces containing vegetation had elevated FCM in the mentioned studies. Gut transit time is faster in bears fed with vegetation diets compared to meat diets [88]. This would tend to cause a decrease in FCM, yet an increase is observed in faeces with vegetation. Therefore, the patterns found in our study probably reflect nutritional impacts on glucocorticoids, rather than effects of only gut retention time or fiber content.

We found a positive correlation between FCM levels and food quality, mostly with FC values (Table 3). Carbohydrate-rich berries are fruiting and abundant when bears are fattening with pre-hibernation hyperphagia, thus adaptive higher levels of corticoids responsible for regulating energy input and storage, help maximizing energy intake. When the glucocorticoids levels rise, it leads to the mobilization of energy reserves. The excreted breakdown products of the stored energy reserves lead to the observed changes in elements and thereby to a correlation with glucocorticoid excretion patterns [7]. Cortisol might also play a role in bone resorption during periods of nutritional stress or affect the amount and type of foods consumed. Its values were found higher in bears eating less salmon in British Columbia, Canada [89] and explained as an adaptive response to food shortage to mobilize fat. Elevated cortisol in hypophagia after denning (in spring) might play a role in minimizing further weight loss by maximizing energy intake from low-fat, herbaceous foods, which are available in spring. Generally, lower levels of FCM in the study area with year-round access to artificial food could be linked to lower diet quality.

The use of faecal constituents is debated and sometimes criticized, especially with respect to unstable ones such as nitrogen [90–92]. Field exposure time appeared to significantly and negatively affect the faecal content of nitrogen in the study by Steyaert et al. [90]. Other studies show that the duration of environmental exposure of faeces in the wild does not seem to affect FN, and so its storage [93]. In diets with secondary plant compounds such as tannins, higher FN values occur because tannins make dietary N partly indigestible. However, in the absence of secondary plant compounds, it should be possible to observe a decrease in FN with a diet rich in N if that diet is at the same time less digestible due to high fibre content. FN should be used with the understanding that it is also a proxy for digestibility but might not be directly related to diet protein content [12,92]. Nevertheless, we used FN, combined with FC as a diet quality proxy, supported by the fact that bears consume highly digestible foods and they tend to avoid tannins in their diet [47]. In addition, artificially provided food items, e.g. wheat and maize, are free of tannins [12]. Thus, FN might be still a useful index to recognize patterns in the diet quality of brown bears, if FN would be stable in field exposure. Any index may have shortcomings; therefore, we advise caution with the interpretation of results when the conditions for the application of FN are not met. As mentioned earlier, there are studies proving both, the environmental instability [e.g. 90] and stability of faecal nitrogen [e.g. 93].

Accurate characterization of diet quality at the population level requires sampling at relatively fine spatial and temporal resolution; infrequent sampling may fail to capture pulses in diet quality that drive population dynamics [94,95], and geographically sparse sampling may fail to include localized hotspots of high forage quality. We believe that, even with opportunistic sampling, our study indicates patterns of food quality and hormonal concentration in areas with and without provision of artificial food. The subpopulation that relies to a great extent on artificial food had lower FN and FC, reflecting lower protein and carbohydrate content in annual diets, and, in general, lower food quality than the one relying exclusively on natural foods. The subpopulation without artificial feeding showed seasonal patterns and the nutritional switch between hypo- and hyperphagia, something that is fundamental to bear´s life and was not observed in the artificially fed subpopulation. Natural seasonal changes in FCM, which are adaptive in species with marked seasonality, were not observed in the area with human-provided subsidies, showing the potential for another possible negative effect of artificial feeding practices. Finally, we showed that faecal indices are a useful tool to gain insights into patterns of diet quality and to assess relationships between food quality and hormonal status accommodating nutritional requirements. Together, this combined approach might enable a better understanding of the physiological adaptations and the nutritional value of brown bear habitats.

## Supporting information

S1 Dataset Faecal cortisol metabolite (FCM; ng/g), faecal nitrogen (FN; %) and faecal carbon (FC; %) values. (XLS)
